# Is it useful to increase dialysate flow rate to improve the delivered Kt?

**DOI:** 10.1186/s12882-015-0013-9

**Published:** 2015-02-14

**Authors:** Marta Albalate, Rafael Pérez-García, Patricia de Sequera, Elena Corchete, Roberto Alcazar, Mayra Ortega, Marta Puerta

**Affiliations:** Servicio de Nefrologia, Hospital Universitario Infanta Leonor, Gran Vía del Este, No 80, 28031 Madrid, Spain

**Keywords:** Dialysate flow rate, KT, Dialysis time, Dialyser design

## Abstract

**Background:**

Increasing dialysate flow rates (Qd) from 500 to 800 ml/min has been recommended to increase dialysis efficiency. A few publications show that increasing Qd no longer led to an increase in mass transfer area coefficient (KoA) or Kt/V measurement.

Our objectives were: 1) Studying the effect in Kt of using a Qd of 400, 500, 700 ml/min and autoflow (AF) with different modern dialysers. 2) Comparing the effect on Kt of water consumption vs. dialysis time to obtain an individual objective of Kt (Ktobj) adjusted to body surface.

**Methods:**

This is a prospective single-centre study with crossover design. Thirty-one patients were studied and six sessions with each Qd were performed. HD parameters were acquired directly from the monitor display: effective blood flow rate (Qbe), Qd, effective dialysis time (Te) and measured by conductivity monitoring, final Kt.

**Results:**

We studied a total of 637 sessions: 178 with 500 ml/min, 173 with 700 ml/min, 160 with AF and 126 with 400 ml/min. Kt rose a 4% comparing 400 with 500 ml/min, and 3% comparing 500 with 700 ml/min. Ktobj was reached in 82.4, 88.2, 88.2 and 94.1% of patients with 400, AF, 500 and 700 ml/min, respectively. We did not find statistical differences between dialysers.

The difference between programmed time and Te was 8′ when Qd was 400 and 500 ml/min and 8.8′ with Qd = 700 ml/min.

Calculating an average time loss of eight minutes/session, we can say that a patient loses 24′ weekly, 312′ monthly and 62.4 hours yearly. Identical Kt could be obtained with Qd of 400 and 500 ml/min, increasing dialysis time 9.1′ and saving 20% of dialysate.

**Conclusions:**

Our data suggest that increasing Qd over 400 ml/min for these dialysers offers a limited benefit. Increasing time is a better alternative with demonstrated benefits to the patient and also less water consumption.

**Electronic supplementary material:**

The online version of this article (doi:10.1186/s12882-015-0013-9) contains supplementary material, which is available to authorized users.

## Background

The National Kidney Foundation’s haemodialysis (HD) practice guidelines (DOQI) recommend a minimum Kt/V of 1.2 and/or a urea reduction ratio (URR) of 65% but HD monitors have incorporated sensors which non-invasively measure the equivalent to urea clearance (K), by using the machine conductivity probes. Calculated K multiplied by the duration of the HD treatment session (t) allows Kt to be obtained, a real measure of dialysis dose, expressed in litres. These sensors enable the dialysis dose to be calculated without additional workload, analytical determinations or cost and quantify the dose received by the patient in each session and in real time [1]. Moreover, K and t are real whereas when Kt/V is used, V refers to the volume and its value based on anthropometric formulas or other methods (bioimpedance) that hamper the evaluation and standardisation of Kt/V. Lowrie et al. proposed Kt as a marker of dialysis dose and mortality [2]. A minimum effective Kt of 40–45 L for women and 45–50 L for men was recommended. In 2005, the minimum Kt dose was individualised according to the body surface area (BSA) [3]. By employing Kt instead of Kt/V we could identify 25.8% of patients that did not reach the recommended minimum Kt, although Kt/V [4] was attained, nevertheless Kt/V remains the method employed by most dialysis units.

The delivered dialysis dose depends on the mass transfer area coefficient of the dialyser (KoA), flow conditions (blood (Qb), Qd and ultrafiltration flow) and dialysis time. Increasing Qd from 500 to 800 ml/min has been recommended to increase the dialysis efficiency and allows shorter treatments. Leypoldt et al. showed that this increase in the Qd results in an increased KoA for urea by 14% (range 3 to 33%, depending on the dialyser model) in an *in vitro* study [5]. They concluded that the improvement was the result of better flow distribution through the dialysate compartment or a decrease in boundary layer resistance to mass transfer on the dialysate side of the membrane. Subsequent clinical publications confirmed this observation [6].

However, in recent years, new dialysers with hollow fibre undulations, spacer yarns and changes in fibre packing density have been developed in order to improve flow distribution through the dialysate compartment [7-10]. A few publications show that increasing Qd no longer led to an increase in KoA [11] but these results are based on Kt/V measurement [12,13], (with the limitations explained above), without comparison between dialysers, and without keeping in mind effective time (Te) or effective Qb (Qbe).

On the other hand, we know that extended treatment times are associated with higher patient survival irrespective of dialysis dose [14-16]. Comparisons between the effect on Kt of increasing dialysis time vs. Qd have not been performed.

The aims of this study are: 1) Studying the effect in Kt of using a Qd of 400, 500, 700 ml/min and autoflow (AF) with different modern dialysers. 2) Comparing the effect on Kt of water consumption vs. dialysis time to obtain an individual objective of Kt (Ktobj) adjusted to body surface.

## Methods

This is a prospective single-centre study with crossover design performed at the Infanta Leonor University Hospital, Madrid, Spain.

To participate in the study subjects were required to be older than 18, have a stable haemodialysis prescription and consent to participate in this study.

The study (identifier: EQdDial-1) was reviewed and approved by the Ethical Committee of Clinical Investigation at Hospital General Universitario Gregorio Marañón (Madrid).

### Study design

The objective of the study was to establish the effect on Kt of increasing the Qd from 400 to 500 and 700 ml/min and the AF™ function.

Monitors and dialysers were identical throughout the study. Twenty-one were dialyzed with AK200™, eight with Fresenius 5008™ and two with Artis™. The membranes used were distributed as follows: nine Xenium H™, eight Xenium M™, seven FX80™ and seven Polyflux 210™. Technical data for the dialysers used are in Table 1.

**Table 1:** **Technical data of the dialysers used**

	**Polyflux 210**	**FXCordiax80**	**XeniumH**	**XeniumM**
Effective surface area (m^2^)	2.1	1.8	2.1	
Packing density (%)	50	60	55	
Shell diameter (mm)	48	41	39	
Fibre outer diameter (μm)	315	220	280	
Number of fibres	12000	13824	11520	
Kuf (ml/h/mmHg)	85	64	82	27
Urea KoA (ml/min)	1450	1263	1975	1450
Hollow-fibre shape	Micro-ondulations			
Membrane component	Polyamix™	Helixone© *plus*	Polynephron™	

All patients were dialysed with conventional bicarbonate haemodialysis as follows:

- Six sessions with Qd at 500 ml/min.
- Six sessions with Qd at 700 ml/min.
- Six sessions with calculated AF. This factor is calculated according to dialyser KoA and Qb [13]. Therapy System™ (Fresenius Medical Care) directly adjusts Qd. AK200 and Artis monitors Qd were programmed according to calculated Qd (Additional file 1).
- Three months later, six sessions with Qd at 400 ml/min.

Treatment time, anticoagulation and Qb were not changed during the study period. Ultrafiltration volumes were set according to clinical needs.

When the 5008™ system measures recirculation, the Qd is temporarily increased to 800 ml/min. Our routine work measures recirculation once a session.

### Parameters collected

Demographic data of 31 patients were collected: sex, age, time in HD and aetiology of end-stage renal disease. Individual Kt objective (Ktobj) was calculated by Kt to body surface area [3].

During the dialysis treatment the following parameters were acquired and recorded directly from the monitor display: effective blood flow rate (Qbe), Qd, effective dialysis time (Te) and measured by conductivity monitoring, final Kt (Ktf).

Calculated data were:

1. Individual medium values of Qbe, Te, Ktf with each Qd
2. Medium clearance per minute Kmin: Ktf*1000/Te (ml/min)
3. Differences of Kt (DifKt) to establish how many patients reach Ktobj: Ktobj minus Ktf

We studied a total of 637 sessions: 178 with 500 ml/min, 173 with 700 ml/min, 160 with AF and 126 with 400 ml/min. Deviations from the prescribed treatment time or blood flow rate or not measuring K due to technical problems were the reasons for session exclusion. Twenty-two patients completed the study with 400 ml/min: one died, two switched HD facility, one was transplanted and five changed HD schedule for clinical reasons (new vascular access, increase of HD time or change of dialysis technique).

### Statistics

Qualitative variables are shown as percentages and quantitative variables as mean (standard deviation) or median (minimum-maximum). Differences between Kt at the different dialysate flow rates were assessed by Student’s paired *t*-test. ANOVA was used to compare quantitative variables as dialyzer or monitor. The chi-squared test was used for qualitative variables. A value of p < 0.05 was considered to be statistically significant.

The analysis was performed with the SPSS computer program, version 15.0.

## Results

### Patients

Thirty one patients were included (eleven female, twenty male) with an average age of 78 (19–92). They had been receiving HD for a median of 19 (3–95) months. The etiology of end-stage renal disease was as follows: 25.8% unknown, 25.8% diabetes, 16.1% vascular, 12.9% interstitial, 12.9% glomerulonephritis, 3.2% reduced renal mass and 3.2% polycystic kidney disease.

Twenty-two patients underwent dialysis three times a week and nine twice a week. In this group, residual renal function (RRF), calculated as medium of urea and creatinine clearance, was higher than 6 ml/min. The programmed dialysis duration was 210′: three patients, 240′: twenty-four patients and 270′: four patients. Finally, twenty-two had arteriovenous fistula and nine tunnelled central venous catheter (CT).

### Dialysis efficiency

The total results appear in Table 2, statistically significant differences in Kt were found. In general, we obtained statistically significant higher Kt and Kmin with larger Qd. Kt increases 4% (400 vs. 500 ml/min) and 2.9% (500 vs. 700 ml/min). AF and Qd = 500 ml/min have similar results, because medium Qd used with AF was 507(52.6) ml/min. No differences in Te or Qbe were found in any case. We did not find statistical differences between dialysers nor monitors

**Table 2:** **HD treatments at different flow rates**

**Qd (n patients)**	**Effective time (min)**	**Effective Qb (ml/min)**	**Kt (L)**	**Kmin**
500 ml/min (n= 31)	**233.4 (6.5)**	**376 (29.6)**	**53.4 (4.6)**	**232.4 (14.4)**
700 ml/min (n= 31)	**233 (7.8)**	**374 (26.4)**	**55 (4.7)** ^c^	**240.7 (13)** ^f^
AF (n= 31)	**235.8 (15)**	**379.6 (29.6)**	**53.2 (3.9)**	**233.8 (13.2)**
400 ml/min (n=22)	**233.3 (5.7)**	**382.3 (18.7)**	**51.3 (2.3)** ^a**,b**^	**219.5 (9.6)** ^d,e^

AF: autoflow. Results with 500, 700 and AF are similar with 31 vs. 22 patients. In comparison with 400 ml/min we only included 22 patients in statistical study (Student’s paired *t*-test).

^a^p<0.04 for Kt 400 ml/min vs. Kt 500 ml/min, ^**b**^p<0.004 for Kt 400 vs. Kt 700 ml/min, ^c^p<0.008 for Kt 700 ml/min vs. Kt 500 ml/min and AF, ^d^p<0.01 Kmin400 vs. Kmin500 and KminAF, ^d^p<0.001 for Kmin400 vs. Kmin700, ^e^p<0.001 for Kmin400 vs. Kmin700, ^f^p<0.0001 for Kmin700 vs. Kmin500 and AF.

We checked whether the obtained Kt for each patient matched the individually calculated value of Ktobj. In Table 3 differences between Ktobj and Ktf are shown. All the patients that did not reach Ktobj had RRF (medium clearance of urea and creatinine > 6 ml/min) with shorter dialysis times.

**Table 3:** **Difference between Ktobjective-finalKt with different Qb (DifKT), results are median(range)**

	**Qd=400**	**Qd=500**	**Qd=700**	**AF**
**DifKT (L)**	4.9 (−4.6-13.9)	7.7 (−3.9-20.6)	6.9 (−1.3-24.4)	6.9 (−1.3-24.4)
**% obj (%)**	82.4	88.2	94.1	88.2

%obj: percentage of patients who reach Ktobjective.

### Dialysis time

A very striking result clearly seen in Table 2 was the waste of time in every HD session. The difference between programmed time and Te was 8′ when Qd was 400 and 500 ml/min and 8.8′ with Qd = 700 ml/min.

Calculating an average time loss of eight minutes/session, we can say that a patient loses 24′ weekly, 312′ monthly and 62.4 hours yearly.

As expected, patients with CT lost more time independently of Qd, although no statistical differences were found.

### Calculations of dialysate consumed

We calculated the volume of dialysate consumed when we used Qd of 400, 500 or 700 ml/min in one patient. Total consumption values are presented in Table 4.

**Table 4:** **Calculated dialysate consumption per patient (acid concentrate relationship 1:45)**

	**Qd-400**	**Qd-500**	**Qd-700**	**Excessive consumption-1**	**Excessive consumption-2**
**Cumulated dialysate 4h session (L)**	**96**	**120**	**168**	**72**	**48**
**L/month**	**1248**	**1560**	**2184**	**936**	**624**
**L/year**	**14976**	**18720**	**26028**	**11232**	**7428**
**Acid concentrate (L) (1:45)**	**2.1**	**2.6**	**3.7**	**1.6**	**1.1**

Excessive consumption-1 is the difference between 700 and 400 ml/min, and excessive consumption 2 between 700 and 500 ml/min.

Based on our results, we calculated required time and dialysate to increase Kt with different Qd using calculated Kmin (Table 2). For example: Kmin with Qd = 400 ml/min was 219 ml/min, if we want to match Kt obtained with Qd = 500 ml/min (2 L higher) we need increase dialysis time 9.1′ and expend 3.6 L more of dialysate (Table 5). We would obtain the same Kt saving 20.4% of dialysate and with and added value: time.

**Table 5:** **Time and dialysate needed to increase Kt**

	**Increasing Kt: 2 L**				**Increasing Kt: 4 L**			
**Qd**	**Time**	**Water (L)**	**Acid (L)**	**Total dialysate (L)**	**Time**	**Water (L)**	**Acid (L)**	**Total dialysate (L)**
400 ml/min	**9.1**	**2.8**	**0.8**	**99.6**	**18.1**	**7.2**	**0.16**	**103.2**
500 ml/min	**8.6**	**3.4**	**0.9**	**124.3**	**17.2**	**8.4**	**0.2**	**128.5**

## Discussion

Our principal result is that increasing Qd from 400 to 500, Kt increases by 4% and from 500 to 700 ml/ml the rise is a 3%. These results are statistically significant but its clinical relevance is questionable. Moreover, Te of HD session is away from the programmed time of eight minutes. So, an equal increase in Kt can be obtained by increasing dialysis time a few minutes with less consumption of dialysate and the unquestionable benefits that longer times have for the patient.

Studies in the 1990s found that KoA and clearance could be increased by increasing Qd (Leypoldt, Ouseph); the result of these studies is the widespread use of Qd of 700 to 800 ml/min to improve clearance. But recognising that poor distribution of flow through the dialysate compartment could create a preferential flow of dialysate in the region external to the bundle with consequent stagnation in the internal region of the haemodialyser, dialysers manufacturers implemented a number of changes in dialysers [17,18]. Some of these technical improvements are: space yarns in the fibre bundle, fibre crossing with a certain angle, fibre undulations, changes in fibre packing density and different flow distributors at the entrance and exit of dialysate compartments. With these approaches, significant increases in solute clearances can be achieved. Three clinical studies have been published studying the effect of increasing Qd with these dialysers. Ward et al. [12] defend the use of a Qd of 600 from 800 ml/min, Alayoud [13] prefers AF because it allows Qd of 400 ml/min whereas Kashiwagi concludes that flow ratio Qb/Qd should be maintained at 1:2 to obtain well-balanced dialysis efficiency [19]. These studies have been performed with different Qb: median of 400, mean of 300 or from 150 to 200 ml/min, respectively, measuring Kt/V and with a single type of dialyser. Also Bhimani et al. [11] studied the dependence of KoA for urea, phosphate and β2-microglobulin on dialysate flow rate in new design dialysers. They concluded that increasing Qd beyond 600 mL/min had only a modest impact on dialyser performance.

Our study provides new relevant information because it is the first time that different dialysate flow rates are compared using a high Qbe. In dialysis facilities using new dialysers, increasing Qd from 500 to 700 ml/min has no meaningful clinical effect as far as Kt is concerned. We do not think that a difference of 1.5 L compensates such greater expenditure of dialysate. Considering that with AF, Qd is calculated based on Qb, we believed that this method could be useful as demonstrated Alayoud et al. Their model predicts the appropriate AF factor that automatically adjusts the Qd according to Qbe and suggests that the value of increasing Qd to compensate for a low vascular access flow is small. In our study we didn’t find any difference because our Qb needs a Qd with AF around 500 ml/min, strengthening our results (additional file 1). Also, using Qd = 400 ml/min individual Ktobj is obtained, although in this case Kt is 2 and 4 L lower than when we used 500 and 700 ml/min, respectively. In any case, only patients with residual renal function did not reach Ktobjt because our treatment is individually adjusted. It is important to emphasize that our results are based on Qbe and Te in contrast to previous studies.

It is comforting to think of water as a renewable resource, but we must also know what limitless exploitation of a resource can lead to. The need to develop more sustainable practices for the management and efficient use of water resources has led to fundamental shifts in awareness and public concern over the past decade. Together we can all do our bit to help reduce water consumption. When we use a Qd = 700 ml/min, we spend a lot water even though no benefit is obtained. Table 4 shows how many liters are saved in only one patient; keeping in mind our dialysis unit with seventy-five patients we waste more than 500000 liters of dialysate. Finally, it is very important to remember that predialysis water treatment uses two liters to produce one liter of dialysate, so that savings are more substancial, nearly one million of liters in our unit.

On the other hand, we know that longer dialysis treatment time is associated with several benefits like better control of anemia, phosphorus level and blood pressure, lower need for erythropoiesis stimulating agents and better overall survival [20]. Taking into account the importance of dialysis time, and in view of our results, we want to emphasize that Te is actually lower than prescribed and that this wasted time could reach 62.4 hours/year, equivalent to 15 HD sessions. Having in mind that dialysis time is an added value, a minor increase of time can reach a higher Kt with a limited consumption of water and concentrates. Therefore, we believe that dialyzing 10′ longer with lower Qd is useful for the patient and at the same time ecological because it involves a more responsible use of water by saving 20% dialysate.

Also, we compare dialysers with different membranes and technical characteristics, all of them far better than older dialyser designs. We were not able to find statistical significant differences between them. This is the first time that different dialysers are compared giving very important information for clinical practice. So, nephrologists can no longer consider the membrane as a single entity. Dialyser may perform very differently depending on the device in which it is employed, with the design regarding performance.

Our results are relevant because they are not based on Kt/V calculated from pre- and postdialysis urea concentrations or V that you can introduce in the monitor. We measure Kt that is an actual measure of HD treatment. We usually calculate Kt individualized according to BSA and this is our individual objective in patients without residual renal function. We would like to draw attention to measuring HD dose by employing Kt alone. We think this is the best method for clinical practice without uncertainty of V and allowing a daily measurement of HD dose without a blood test.

The principal limitation of our study is that the number of patients included is small but on the other hand, we think our results are improved because the number of dialysis sessions is high and we use Student’s paired *t*-test, comparing each patient with himself. Also, our study has been performed by a single dialysis unit but we think our results can be extrapolated to all units that work in similar conditions. Finally, comparison with Qd of 400 ml/min has been carried out only in one group of the patients, because we realised that this information is absent and it could be very useful. Although the number of patients is lower the design of the study allows us to confirm our results.

## Conclusions

Our data suggest that increasing dialysate flow to 400 ml/min for these dialysers offers a limited benefit. If we do not improve HD efficiency, water saving is important to protect the environment and to meet current and future human demand. It is important to keep in mind that in order to produce one litre of dialysate in reverse osmosis two litres of water are needed. So, increasing time is a better alternative with demonstrated benefits for the patient and less water consumption.

## Additional files

Additional file 1:
**Simplified formula, where we consider that Qb is the blood flow rate (ml/min) and KoA is the mass transfer area coefficient for urea (millilitres per minute).** This simplified equation will lead to a small overestimation of the real Autoflow factor.

## Abbreviations

AF: Autoflow
HD: Hemodialysis
Kmin: Medium clearance per minute
KoA: Mass transfer area coefficient
Ktf: Final Kt
Ktobj: Individual objective Kt
Qbe: Effective blood flow rate
Qd: Dialysate flow rate
Te: Effective dialysis time
