# Supplementary figures and images for: Is it useful to increase dialysate flow rate to improve the delivered Kt?
Source: BMC Nephrol. 2015 Feb 14;16:20. doi: 10.1186/s12882-015-0013-9 (PMC4369100; doi:10.1186/s12882-015-0013-9)

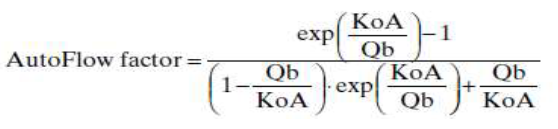


| **Dialyser** | **Qb** | **AF** | **Qd calculated** |
| --- | --- | --- | --- |
| **FX80** | 300 | **1.3** | **400** |
| 350 | **1.3** | **450** |
| 400 | **1.3** | **525** |
| 450 | **1.4** | **650** |
| **Polyflux210**  **XeniumM** | 300 | **1.3** | **400** |
| 350 | **1.3** | **450** |
| 400 | **1.3** | **525** |
| 450 | **1.4** | **650** |
| **XeniumH** | 300 | **1.2** | **375** |
| 350 | **1.2** | **425** |
| 400 | **1.2** | **500** |
| 450 | **1.3** | **600** |

Supplement: Additional file 1: — Simplified formula, where we consider that Qb is the blood flow rate (ml/min) and KoA is the mass transfer area coefficient for urea (millilitres per minute). This simplified equation will lead to a small overestimation of the real Autoflow factor. [file 12882_2015_13_MOESM1_ESM.doc]
